# Comparison of efficacy and safety of combination therapy with statins and omega-3 fatty acids versus statin monotherapy in patients with dyslipidemia: A systematic review and meta-analysis

**DOI:** 10.1097/MD.0000000000013593

**Published:** 2018-12-14

**Authors:** Hye Duck Choi, Seung Min Chae

**Affiliations:** College of Pharmacy, Yeungnam University, Gyeongsangbuk-do, Republic of Korea.

**Keywords:** dyslipidemia, meta-analysis, omega-3 fatty acids, statins, systematic review

## Abstract

**Objective:**

Dyslipidemia is a major risk factor for the development of cardiovascular disease. Both statins and omega-3 fatty acids demonstrate beneficial effects on lipid concentrations. The goal was to evaluate the safety and efficacy of combination therapy with statins and omega-3 fatty acids.

**Methods:**

We performed a systematic review and meta-analysis of published data to compare the safety and efficacy of combination therapy with statins and omega-3 fatty acids versus statin monotherapy in patients with dyslipidemia. Six articles were assessed in the present meta-analysis (quantitative assessment) and qualitative assessment.

**Results:**

In terms of efficacy, the combination treatment afforded a significantly greater reduction in total cholesterol/high-density lipoprotein cholesterol than statin alone did [standard difference in means = −0.215; 95% confidence interval (CI) −0.359–−0.071]. However, there was no significant difference in low-density lipoprotein (LDL) cholesterol between the 2 groups. Qualitative assessment of other lipid parameters was performed. Combination therapy with statins and omega-3 fatty acids was generally more effective on lipid concentration than statin monotherapy. In terms of safety, there were no significant differences in total adverse events between the 2 groups. Gastrointestinal adverse events were found to be significantly increased in patients receiving combination therapy using the fixed-effects model (relative risk = 0.547; 95% CI 0.368–0.812).

**Conclusions:**

We suggest that combination therapy with statins and omega-3 fatty acids enhances lipid profile, except LDL cholesterol, compared with statin monotherapy. Nevertheless, statin and omega-3 fatty acid combination should be cautiously recommended, taking into account the clinical importance of LDL cholesterol and safety issues associated with their concomitant use.

## 1 Introduction

Dyslipidemia is a clinical condition characterized by elevated total and low-density lipoprotein (LDL), high triglycerides, and low high-density lipoprotein (HDL) cholesterol. It is one of the major risk factors for the development of coronary heart disease. The condition should therefore be controlled to prevent primary or recurrent cardiovascular events. Several pharmacotherapeutic options are available to control lipid concentrations and reduce cardiovascular death and total mortality.

Statins are the initial treatment choice for dyslipidemia in most patients. They are hydroxymethylglutaryl coenzyme A reductase inhibitors and have potent total and LDL cholesterol-lowering effects.^[1]^ Combination therapy may be considered in patients not responding to statin monotherapy. In such cases, fibrates, niacin, bile acid resins, or ezetimibe are used concomitantly or alone; however, the patients should be monitored closely for the development of adverse effects and drug interactions.

Omega-3 polyunsaturated fatty acids are also used as an alternative therapy to regulate abnormal lipid concentrations. The efficacy of fish oil supplementation containing high omega-3 fatty acids on lipid metabolism is mediated through the reduction in very low-density lipoprotein (VLDL) production and suppression of VLDL and apolipoprotein B (apoB).^[2–4]^ Lipid profile changes, including reduction in cholesterol and triglycerides, by omega-3 fatty acids have been reported in normal volunteers and patients with hyperglyceridemia.^[5,6]^ Therefore, omega-3 fatty acids may be useful in patients with elevated triglyceride levels; however, the clinical role of omega-3 fatty acids is not well defined. Moreover, the safety and efficacy of combination therapy with omega-3 fatty acids and statins have not been evaluated adequately.

Recently, several clinical trials have shown that combination therapy with statins and omega-3 fatty acids improved lipid profiles and prevented cardiovascular events.^[7–12]^ Nevertheless, the results of trials comparing the efficacy of statin monotherapy versus combination therapy with omega-3 fatty acids have not yet been integrated into clinical decision making. In addition, the safety of combination therapies remains controversial.

Therefore, we performed a systematic review and meta-analysis to determine the changes in lipid concentrations and the number of adverse events in the reported studies by comparing the effects of statin monotherapy versus combination therapy with statins and omega-3 fatty acids.

## 2 Methods

### 2.1 Search strategy

We searched for published articles comparing the lipid-lowering effects and safety of statins and omega-3 fatty acids in patients with dyslipidemia. We searched online databases, including MEDLINE (OVID and PubMed) and the Cochrane Library. The search terms were combinations of the following PubMed MeSH terms and related text terms: *hydroxymethylglutaryl-CoA reductase inhibitors*, *statins*, *omega-3 polyunsaturated fatty acids*, *omega-3*, *omega-3 fatty acids*, *dyslipidemia*, *hypercholesterolemia*, and *hyperglyceridemia*. The bibliographies of retrieved articles and relevant reviews were searched to identify additional eligible studies. We did not impose any publication or language limitations. The search was completed on January 15, 2017.

### 2.2 Study selection

Two authors independently reviewed and selected studies for inclusion in the systematic review. The inclusion criteria were as follows: the study was a randomized clinical trial; compared the efficacy and safety of fibrates and statins; included measurements of lipid profiles; and described a number of adverse events. Any disagreement in terms of inclusion of an article for evaluation was resolved by discussion. If a trial was described in more than one report, we extracted data from the most complete account and used the other publications only to clarify those data.

### 2.3 Data extraction

Detailed reviews of full-text articles were performed independently by 2 authors. The following data were extracted from each study: the first author's surname; the year of publication; the country in which the work was performed; the number of participants; patient characteristics (type of dyslipidemia, sex, and age); the treatment given (regimen and period); and changes in serum lipid concentrations.

### 2.4 Risk of bias within studies (quality assessment)

The methodological quality of each trial was evaluated by 2 authors with the aid of the Jadad scale.^[13]^ The Jadad scale evaluates randomized controlled trials using the following 5 indicators: an adequate description of how randomization was achieved; the appropriateness of the randomization method; an adequate account of how the investigators were double blinded; the appropriateness of the double-blinding method chosen; and details on patient withdrawal and dropout. A score >3 was considered to reflect high-quality work. Any disagreement between the 2 authors was resolved by discussion.

### 2.5 Outcome measures

#### 2.5.1 Analysis of efficacy

In terms of evaluation of efficacy, the endpoints of analysis were changes in lipid concentrations, including changes in total cholesterol, LDL cholesterol, HDL cholesterol, triglycerides, and other related lipid concentrations. For the meta-analysis, the change in LDL cholesterol and total cholesterol/HDL cholesterol ratio, respectively, was calculated as the difference between the baseline and final measurements performed in each study group. Each mean change, with a 95% confidence interval (CI), was calculated to allow assessment of the lipid-lowering effects of statin monotherapy or combination therapy with omega-3 fatty acids.

#### 2.5.2 Analysis of safety

To evaluate treatment safety, we counted the total number of adverse events and gastrointestinal adverse events in each study group, and compared these values between treatments. Relative risk (RR) values and 95% CIs were calculated to compare the frequencies of adverse events associated with the use of statin monotherapy or combination therapy with omega-3 fatty acids.

### 2.6 Risk of bias across studies and statistical analysis

Study heterogeneity was assessed using the χ^2^ test (employing Q statistics) and quantified by calculating *I*^2^ values.^[14]^ A fixed-effects model (the Mantel-Haenszel method) was used in the analysis.^[15]^ The results were compared to those yielded by a random-effects model (the Der Simonian-Laird method).^[16]^

Sensitivity analyses were performed by excluding the contribution of each study to the meta-analysis data in turn. The potential existence of publication bias was examined using Begg and Egger tests.^[17,18]^

All statistical analyses were performed using the Comprehensive Meta-analysis Software version 2 (CMA 26526; BioStat, Englewood, NJ). All statistical tests were 2 sided and a value of *P* < .05 was considered to indicate statistical significance.

### 2.7 Others

This systematic review was performed based the previously reported articles. Therefore, any ethical review or approval is not applicable.

## 3 Results

### 3.1 Study quality and characteristics

**Figure 1:**
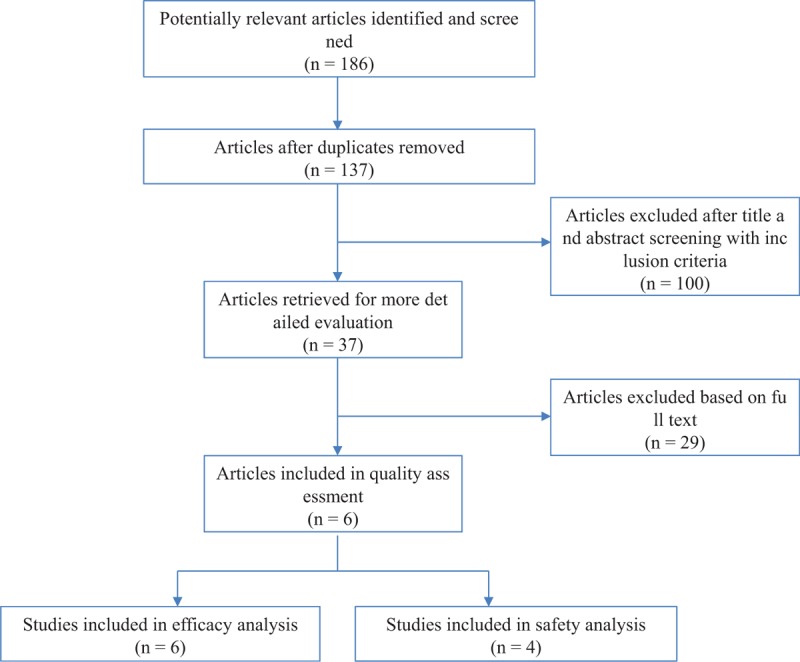
PRISMA diagram of the process of selecting relevant studies.

One hundred eighty-six articles were identified by the literature search. After removal of duplicates, the titles and abstracts of 137 articles were screened. Of these, 100 articles were excluded, and the full texts of the remaining 37 articles were assessed in terms of eligibility. A further 29 articles were excluded, and data from the remaining 6 articles were included in the present systematic review (Table 1). Figure 1 shows the study selection flow chart according to PRISMA.^[19]^ Using the Jadad system, 2 studies were classified as low quality (a score of ≤2) and 4 studies as high quality (scores of ≥3).

**Table 1:**
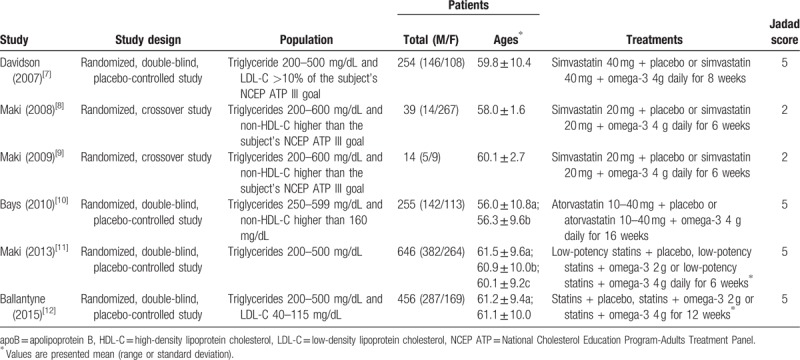
General characteristics of included studies.

### 3.2 Meta-analysis of efficacy

#### 3.2.1 LDL cholesterol

**Figure 2:**
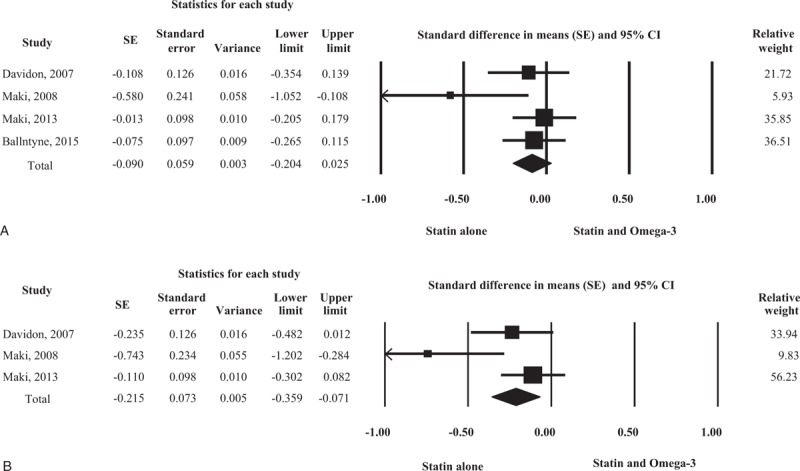
Forest plot of efficacy. Changes in low-density lipoprotein (LDL) cholesterol (A) and total cholesterol/high-density lipoprotein, (HDL) cholesterol ratio (B) compared between statin monotherapy and combination therapy with omega-3 fatty acid.

Four studies measured the changes in LDL cholesterol in 589 patients treated with statin alone and 581 patients treated with statin plus omega-3 fatty acid (Fig. 2A). There were no significant differences in LDL cholesterol changes between the 2 groups, and re-analysis using a random-effects model yielded the same result.

#### 3.2.2 Total cholesterol/HDL cholesterol ratio

Three studies assessed the changes in *total cholesterol/HDL cholesterol ratio* in 382 patients treated with statin alone and 368 patients treated with statin plus omega-3 fatty acid (Fig. 2B). The combination treatment afforded a significantly greater reduction in *total cholesterol/HDL cholesterol* than statin alone did. Data re-analysis using a random-effects model also revealed a significant difference between the 2 treatments.

### 3.3 Qualitative assessments of other lipid parameters

Lipid parameters, except LDL cholesterol and total cholesterol/HDL cholesterol ratio, are presented in Table 2.

**Table 2:**
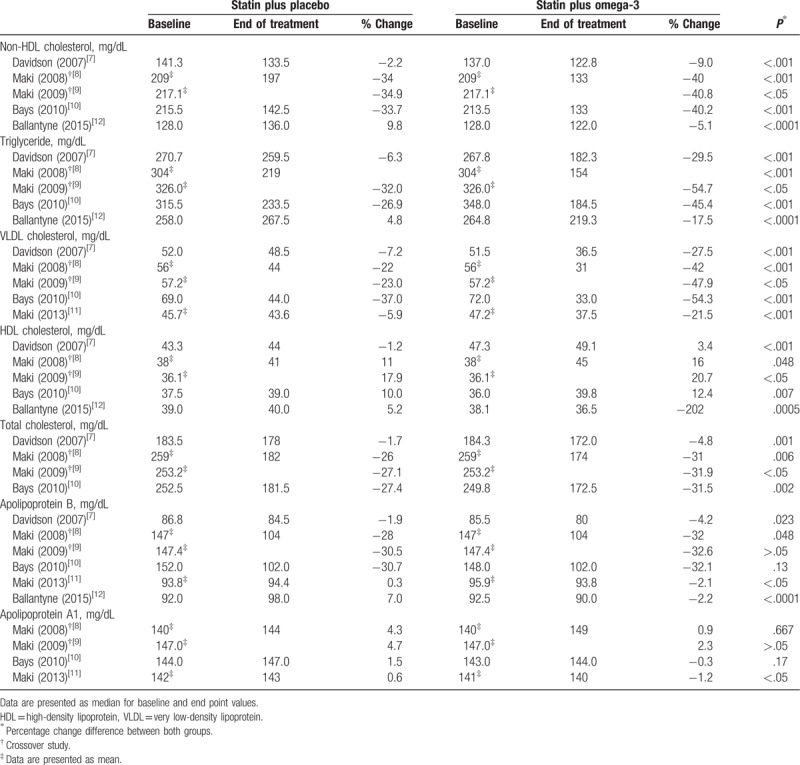
Qualitative assessments of other lipid parameters.

### 3.4 Meta-analysis of safety

#### 3.4.1 Total adverse events

**Figure 3:**
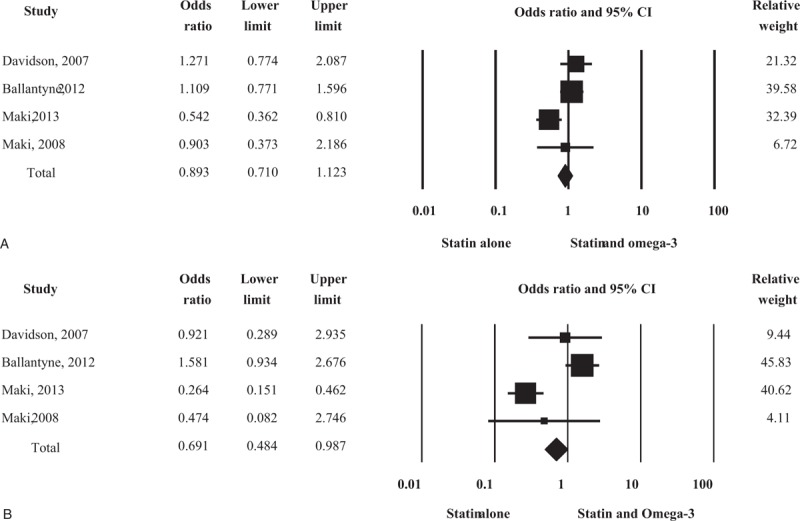
Forest plot of safety. Total adverse events (A) and gastrointestinal adverse events (B) compared between statin monotherapy and combination therapy with omega-3 fatty acids. CI = confidence interval.

In total, 4 studies described adverse events, including treatment-related side effects. Two hundred fifty-two adverse events occurred in 620 patients (40.6%) treated with statin alone, and 265 adverse events occurred in 611 patients (43.4%) treated with statin plus omega-3 fatty acid (Fig. 3A). There were no significant differences in total adverse events between the 2 groups. Re-analysis using a random-effects model yielded the same result.

#### 3.4.2 Gastrointestinal adverse events

Sixty-seven gastrointestinal adverse events occurred in 620 patients (10.8%) treated with statin alone and 95 occurred in 611 patients (15.5%) treated with statin plus omega-3 fatty acid (Fig. 3B). The gastrointestinal adverse events were significantly increased in patients who received combination therapy with omega-3 fatty acid. However, re-analysis using a random-effects model yielded no significant results (RR = 0.667; 95% CI 0.283–1.996).

### 3.5 Sensitivity analyses and publication bias

Sensitivity analysis was performed by recalculating all findings after data from each study included in the meta-analysis were omitted in turn. The findings were not altered significantly throughout this process (data available upon request). We also evaluated publication bias. The funnel plots were not obviously asymmetric. The results of Begg rank-correlation test and Egger regression test are shown in Table 3. No publication bias was observed for any tested comparison.

**Table 3:**
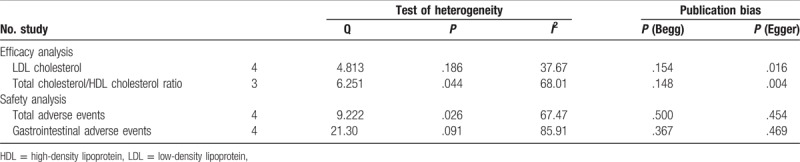
Test of heterogeneity and publication bias.

## 4 Discussion

### 4.1 Summary of the main results

We performed a systematic review and meta-analysis to evaluate the safety and efficacy of statin monotherapy and combination therapy with statin and omega-3 fatty acid in patients with dyslipidemia. First, we found that changes in lipid concentration differed significantly between the 2 drug regimens. Combination therapy with statin and omega-3 fatty acid afforded greater reduction in total cholesterol/HDL cholesterol ratio. Second, treatment with statin plus omega-3 fatty acid was associated with a significant increase in the number of gastrointestinal adverse events, but not in total adverse events.

We performed the qualitative assessment of other lipid concentrations owing to lack of adequate number of studies or different data types to perform a meta-analysis (Table 2). Five studies were included to evaluate non-HDL cholesterol, triglycerides, and HDL cholesterol.^[7–10,12]^ In all studies, the combination treatment afforded significantly greater reductions in non-HDL cholesterol and triglycerides than treatment with statin alone did. In addition, the combination treatment was associated with significant increases in HDL cholesterol. Five studies evaluating VLDL cholesterol showed significant cholesterol-lowering effects by the combination treatment.^[7–11]^ Moreover, the combination treatment was found to be more effective in reducing total cholesterol than statin monotherapy in the 4 included studies.^[7–10]^ Six studies were included to evaluate apoB, and 2 of the studies showed no significant difference between the 2 groups.^[7–12]^ A significant association between combination treatment and apolipoprotein A1 increase was shown in 1 of the 4 included studies.^[11]^ Overall, the results of qualitative assessment showed that combination therapy with statin and omega-3 fatty acid was generally more effective in regulating lipid concentration than statin monotherapy.

LDL cholesterol is clinically the most important measurement in a lipid profile, and it is closely related to cardiovascular disease or death.^[20,21]^ Based on the present meta-analysis, no significant difference in LDL cholesterol levels between statin monotherapy and combination therapy with omega-3 fatty acid was inferred; however, the offsetting effect of omega-3 fatty acid on LDL cholesterol cannot be completely excluded. This finding is in agreement with the results of previous studies in which omega-3 fatty acid consumption was reported to be associated with mild increases in LDL cholesterol in patients with hypertriglycemia and dyslipidemia.^[5,6]^ This change can be explained, at least in part, by the increased rate of conversion of VLDL to LDL particles.^[2–4]^

The results of the meta-analysis by Sethi et al^[22]^ are noteworthy. The meta-analysis was performed to estimate the total and cardiovascular mortalities in participants receiving omega-3 fatty acid, followed by a metaregression of dose, docosahexaenoic acid/eicosapentaenoic acid (DHA/EPA) ratio, duration of treatment, and use of lipid-lowering/statin therapy in control group. The results showed that omega-3 fatty acid had no effect on total mortality (RR = 0.96; 95% CI, 0.92–1.01), but it reduced cardiovascular mortality (RR = 0.93; 95% CI, 0.87–0.98). In addition, lower control group statin use and higher DHA/EPA ratio was associated with higher reduction in total mortality. In other words, statin use may mitigate the positive effects of omega-3 fatty acid. Further research or clinic trials can provide more evidence on the influence of combination therapy with statin and omega-3 fatty acid on LDL cholesterol.

In the meta-analysis on safety, the incidence of gastrointestinal adverse events was slightly higher in patients receiving statin and omega-3 fatty acid combination therapy. Statin or omega-3 use is associated with several side effects ranging from mild to severe. Gastrointestinal problems are frequently caused by treatment with statins and omega-3 fatty acid alone, and their concomitant use may increase the risk of side effects significantly. Therefore, the adverse events should be monitored more intensively in patients receiving combination treatment.

### 4.2 Strengths and limitations

There are certain limitations to our present meta-analysis. First, only a small number of relevant studies were included and data were inadequate. Second, our analysis was based on previous reports, which were not necessarily complete or accurate, and we were unable to analyze data, including sex, ages, duration, or other factors. Third, the results based on statistical analysis could be partially different from the evaluations of safety or efficacy in individual patients.

Despite these limitations, to the best of our knowledge, this is the first study to compare the safety and efficacy of statin treatment alone versus combination therapy with statin and omega-3 fatty acid in patients with dyslipidemia. We derived accurate estimates of the clinical efficacies of the 2 types of treatment. Our data have greater statistical power than the data subsets of the reports included in the meta-analysis.

## 5 Conclusions

Overall, we suggest that combination therapy with statins and omega-3 fatty acid enhances the lipid profile, except LDL cholesterol, when compared with statin monotherapy. However, controlling LDL cholesterol levels is important for preventing cardiovascular diseases and related deaths. Furthermore, safety issues with the concomitant use of statins and omega-3 fatty acid should be considered. Thus, combination therapy with statins and omega-3 fatty acid should be cautiously recommended after assessing the benefits and risks.

## Author contributions

The contributions of the authors to the manuscript are as follows. Choi HD: study design, data collection, data analyses, coordination, and writing the manuscript; S.M.C.: study design, data collection, data analyses, and reviewing the manuscript. All authors read and approved the final manuscript.

**Conceptualization:** Hye Duck Choi.

**Data curation:** Hye Duck Choi, Seung Min Chae.

**Formal analysis:** Hye Duck Choi, Seung Min Chae.

**Funding acquisition:** Hye Duck Choi.

**Investigation:** Hye Duck Choi, Seung Min Chae.

**Methodology:** Hye Duck Choi.

**Project administration:** Hye Duck Choi.

**Resources:** Hye Duck Choi.

**Software:** Hye Duck Choi.

**Supervision:** Hye Duck Choi.

**Validation:** Hye Duck Choi.

**Visualization:** Hye Duck Choi.

**Writing – original draft:** Hye Duck Choi.

**Writing – review and editing:** Hye Duck Choi.

Hye Duck Choi orcid: 0000-0003-2292-6827.
